# Septic Arthritis Complicating a Gout Flare: Report of Two Cases and Review of the Literature

**DOI:** 10.31138/mjr.33.1.75

**Published:** 2022-03-31

**Authors:** Prokopios Tzanis, Kalliopi Klavdianou, Argyro Lazarini, Evangelos Theotikos, Alexia Balanika, Antonis Fanouriakis, Antonia Elezoglou

**Affiliations:** 1 Department of Rheumatology, “Asklepieion” General Hospital, Athens, Greece,; 2 Department of Radiology, “Asklepieion” General Hospital, Athens, Greece

**Keywords:** septic arthritis, gout, Streptococcus agalactiae, IL-1 inhibitors

## Abstract

**Objective:**

To highlight potential pitfalls in diagnosis and management of patients with concomitant gout and septic arthritis.

**Methods:**

Presentation of two patients with concomitant gout and septic arthritis, the latter caused by *Streptococcus agalactiae* and *Staphylococcus aureus*, in one patient each. We also reviewed the English language literature on PubMed for similar cases.

**Results:**

Data on concurrent gout and septic arthritis is limited. Three case series of 14, 25, and 30 patients each where identified. The coexistence of septic arthritis and gout is an infrequent condition. Clinical appearance of the two diseases may be very similar and the presence of monosodium urate (MSU) crystals *per se* cannot exclude infection. On the other hand, patients with chronic tophaceous gout are prone to infection of MSU tophi and the development of biofilms on the latter may render the eradication of microbes particularly difficult. Vice versa, persistent activation of the immune system fuelled by the infection, together with prolonged hospitalisation and immobilisation, may increase the risk for a gout flare, thus initiating a vicious cycle.

**Conclusion:**

In patients with gout, a high index of suspicion for infection is needed by treating physicians, because septic arthritis is a medical emergency which can lead to rapid joint destruction.

## INTRODUCTION

Septic arthritis is an emergency situation potentially leading to cartilage destruction, severe disability, and increased mortality.^1,2,3^ A high index of suspicion to allow early diagnosis and treatment is necessary. On the other hand, gout is the most common form of inflammatory arthritis, caused by excess accumulation of monosodium urate (MSU) crystals in joint fluid, cartilage, tendons, and other sites. In severe, persistent forms or in patients with contraindication or intolerance to standard of care treatment, treatment with IL-1 blockers is often considered.^10,11^ The coexistence of gouty and septic arthritis is a rare medical condition that can frequently be misdiagnosed, leading to adverse sequelae. To this end, prompt synovial fluid aspiration and examination is of utmost importance to discriminate between these two conditions.^6,13^

Herein, we describe two patients who were initially referred to our Department for a possible gout flare and were diagnosed with septic arthritis complicating gout. We review the literature related to the coexistence of these two conditions, highlighting potential pitfalls in their diagnosis and management.

## CASE 1

A 57-year-old man with a past medical history of tophaceous gout and alcohol abuse was referred to the Emergency Department of our hospital, due to severe pain and swelling of the right knee and right ankle, of two weeks’ duration. His medication included nebivolol 5mg and an irregular use of allopurinol, colchicine and NSAIDs. Ten days prior to the visit in the Emergency Department, he was treated by a rheumatologist in another facility with glucocorticoids and indomethacin.

**Figure 1.**
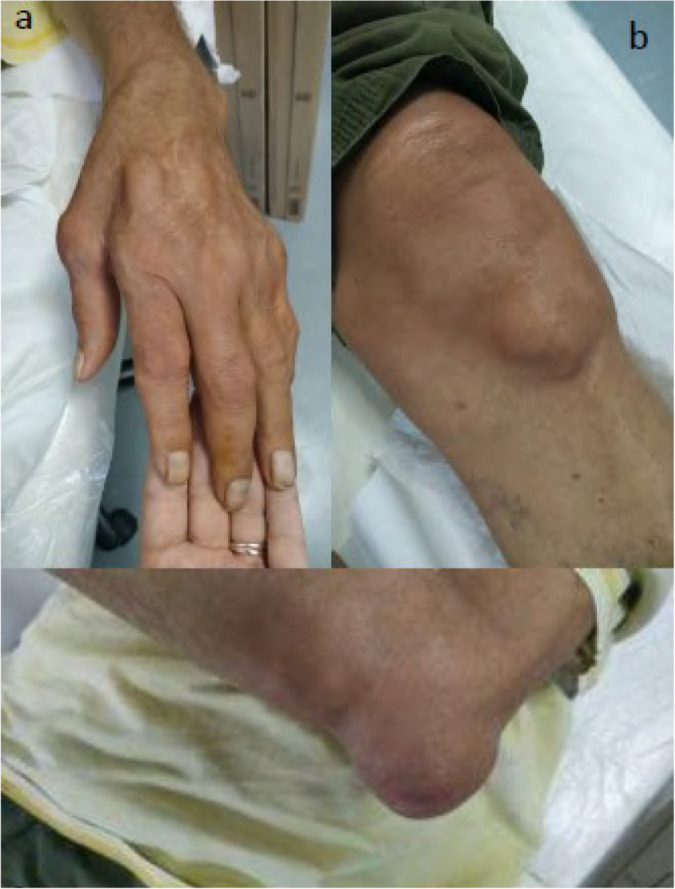
Clinical images of patient #1 at presentation. Multiple tophi are present in the patient’s **(A)** hand, **(B)** knee, and **(C)** elbow.

On examination in our hospital, the patient was febrile with a body temperature of 38°C, normal vital signs, and oxygen saturation. Physical examination revealed active arthritis of the right knee and ankle with affected range of motion, as well as existence of numerous tophi in the hands, wrists, elbows, and knees (**Figure 1**). The patient was unable to walk on his own. Laboratory tests showed neutrophilic leucocytosis (16,700 cells/μl), profound hyperuricemia (12.3 mg/dl) and a raised C-reactive protein (CRP, 193 mg/l, normal range < 5). Chest ray and electrocardiography were unremarkable.

Aspiration of the right knee revealed purulent synovial fluid with 112,000 cells/μL. On microscopy, abundant MSU crystals were evident, but microorganisms were also observed. A Gram’s stain was positive for Gram-positive cocci and synovial fluid culture revealed *Streptococcus agalactiae*. Following the Gram’s stain and pending the results of synovial fluid culture, empiric treatment for septic arthritis was initiated with intravenous daptomycin and levofloxacin, which was tailored to penicillin and levofloxacin 750mg/d IV upon receiving the culture results. Orthopaedic examination consulted against surgical or arthroscopic drainage.

**Figure 2.**
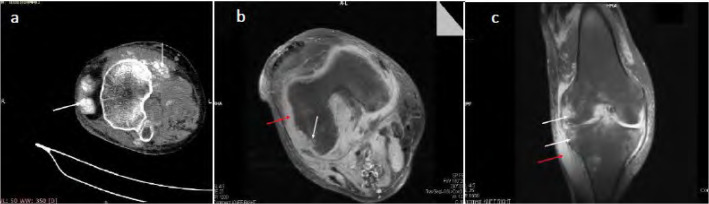
**(a)** CT scan of right knee (axial plane) showing extraarticular and intra-articural calcified lesions (tophi) (white arrows). **(b)** MRI of right knee (axial plane) showing cortical erosions at the articular surface of the lateral femoral condyle (white arrow) and a joint effusion (red arrow). **(c)** MRI of right knee (coronal plane) showing cortical erosions at the articular surface of the lateral femoral and tibial condyle (white arrows) and a joint effusion (red arrow).

On day 2 of hospitalisation, aspiration of the left elbow was performed due to new onset arthritis, again revealing puru-lent joint fluid with additional MSU crystals. *Streptococcus agalactiae* was again isolated; accordingly, knee and elbow arthrocentesis were repeated daily until sterilization of synovial fluid cultures. A computed tomography (CT) scan of the right knee and ankle was performed, revealing multiple well-defined corticated erosions of lateral femoral condyle, tibia, fibula, calcaneus, and tarsal bones and high attenuation intraarticular or extraarticular soft tissue masses (**Figure 2A**), consistent with tophi. Magnetic resonance imaging (MRI) of the knee showed intra-articular fluid collection and oedema of adjacent muscles, without evidence of osteomyelitis (**Figure 2 B,C**) Transthoracic and transoesophageal echocardiography were negative for vegetations and blood cultures were sterile, ruling out bacterial endocarditis.

On day 5 of hospitalisation, the patient experienced an episode of acute lower gastrointestinal bleeding with haematochezia and resulting severe anaemia (Hb 6,8 g/dl), necessitating transfusions of red bloods and fresh frozen plasma. Urgent endoscopy attributed the bleeding to ascending colon ulcers and the patient’s condition was gradually stabilised.

During the course of the patient’s hospitalisation, in parallel to a gradual clinical improvement of the arthritis in the lower limb and sterilisation of synovial fluid cultures, the patient developed acute polyarthritis of both hands, left wrist and right elbow. A diagnosis of acute gout was suspected and therapy with prednisone 30 mg/day was initiated. Due to suboptimal response and persistent intensity of symptoms, we decided to administer interleukin-1 (IL-1) inhibition with anakinra, which resulted in a remarkable clinical response. Following approval from the regulatory authorities, a single dose of canakinumab 150 mg was subsequently administered, leading to further improvement of the patient’s polyarthritis. The patient was finally discharged following the completion of 6 weeks of antibiotic therapy, with hypouricemic treatment with febuxostat and on-demand use of canakinumab. Rigorous physiotherapy was also recommended due to lower limb muscular atrophy, owing to prolonged immobilisation.

## CASE 2

A 77-year-old man with a past medical history of gout was referred to our Department from another hospital, where he was hospitalised for 4 days due to a painful and swollen right ankle. Prior to referral, he was treated as a possible gout flare with glucocorticoids, with no improvement of his symptoms. The patient’s symptoms had started 3 weeks prior to admission, when he was initially examined by an orthopaedic surgeon and treated with colchicine and non-steroidal anti-inflammatory drugs (NSAIDs); intraarticular glucocorticoids had also been administered in the right ankle.

**Figure 3.**
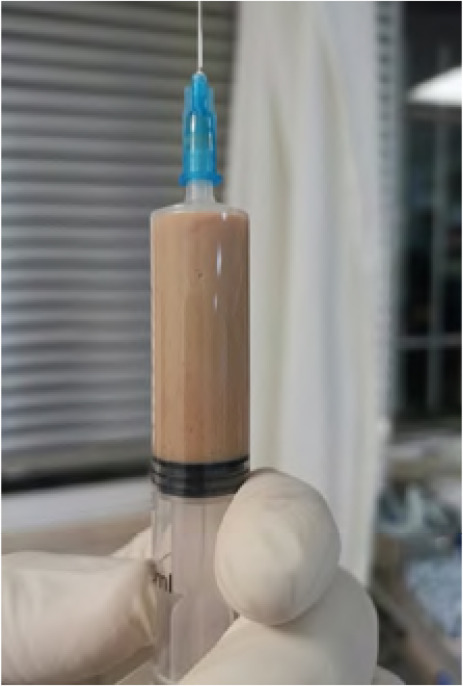
Purulent synovial fluid aspirated from the ankle of patient #2.

**Figure 4.**
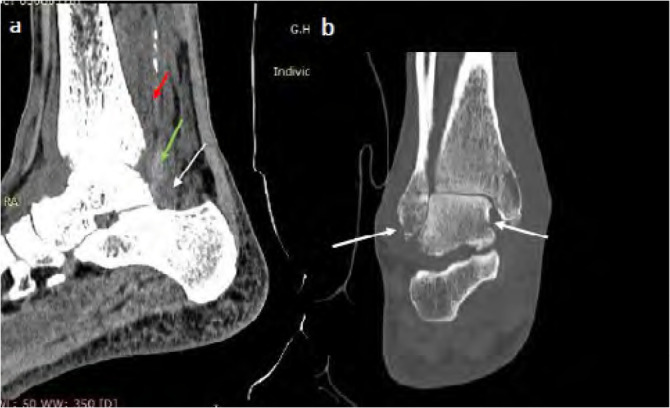
**(A)** CT scan of right ankle (sagittal reformatted image) showing subcutaneous tissue and muscle oedema (red arrow), posterior tibiotalar joint fluid (white arrow) and periarticular tophi (green arrow). **(B)** CT scan of the right ankle (coronal reformatted image, bone algorithm), demonstrating several erosions of the articular surfaces of the tibia, fibula, and talus.

On examination in our Department, the patient was afebrile; physical examination revealed profound arthritis of the right ankle, with erythema and swelling. Laboratory tests were remarkable for significant neutrophilic leucocytosis (30,400 cells/μl), elevated CRP (136 mg/l, normal range < 5) and ESR levels (135 mm/hr), and a urate level of 8.4 mg/dl. Aspiration of the right ankle was performed, which yielded purulent synovial fluid (**Figure 3**), along with additional presence of MSU crystals on direct microscopy. Gram’s stain revealed Gram-positive cocci and methicillin-sensitive *Staphylococcus aureus* (MSSA) was isolated from synovial fluid culture. Initial empiric treatment with intravenous daptomycin and piperacillin/tazobactam was initiated, subsequently tailored to cloxacillin and moxifloxacin 400mg/d IV, following culture results. Glucocorticoids were tapered down to full discontinuation. Blood cultures were also positive for MSSA, but transthoracic and transoesophageal echocardiography did not show evidence of bacterial endocarditis. A CT scan of the right ankle revealed subcutaneous tissue and muscle swelling, significant effusion in the ankle joint (**Figure 4A**), as well as several erosions at articular surfaces of tibia, fibula, talus, and calcaneus, with periarticular high attenuation soft tissue deposits (tophi). findings more compatible with sequelae of chronic gout. (**Figure 4B**)

**Figure 5.**
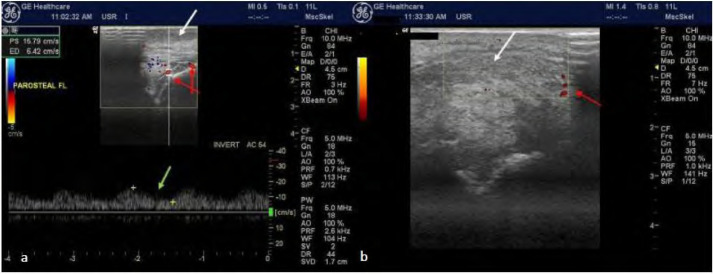
**(A)** CDUS of the right ankle (sagittal image of medial malleolus) showing, subcutaneous tissue oedema and posterior tibialis tendon swelling and paratendon inflammation (tenosynovitis) with hyperaemia and parosteal arterial flow. **(B)** CDUS of the right ankle (sagittal image of medial malleolus) 3 weeks later showing presence of mild muscle hyperaemia and significant improvement of ultrasonographic findings.

Following orthopaedic consultation, the patient underwent arthroscopic lavage on day 12 of hospitalization; synovial fluid with a purulent, but also “chalk-like” appearance was evident during arthroscopy. The patient gradually improved, with a parallel drop in CRP and ESR values. Nevertheless, due to persistent, painless calf and leg oedema, on day 20 a Colour Doppler Ultrasound (CDUS) was performed, which demonstrated subcutaneous oedema and hyperechogenity in soleus and posterior tibialis muscles and tendon, with hyperaemia (pyomyositis) and paratendon inflammation (tenosynovitis), tibiotalar joint synovial fluid, without cortical discontinuity and periosteal flow, all findings suggestive of a soft tissue infection without bone involvement (**Figure 5A**). Following consensus by the rheumatology, orthopaedics and infectious disease units, and given the patient’s improving clinical picture (absence of pain, falling CRP), it was decided to continue conservative treatment with IV antibiotics until 6 weeks were completed, followed by prolonged PO treatment. Follow-up CDUS after 1 and 3 weeks showed significant improvement of both articular and muscular involvement (**Figure 5B**).

During the course of the patient’s hospitalisation, new appearance of arthritis in the left metatarsophalangeal joints and midfoot was consistent with a new gout flare, and short-course PO glucocorticoids were re-administered. Finally, following 6 weeks of hospitalization and IV antibiotic therapy, the patient was discharged from hospital, with the recommendation to continue PO antibiotics (levofloxacin 750mg bid and rifampin 300mg bid) for a total of 12 weeks, hypouricemic treatment with allopurinol 100mg/day and physiotherapy at home.

## DISCUSSION

Septic arthritis is a rheumatologic emergency, as it can lead to joint destruction within days,^1,2,3^ and is accompanied by significant mortality ranging from 7–15%.^1,2^ Risk factors for septic arthritis are pre-existing joint diseases, including gout, and comorbidities, such as rheumatic conditions, alcohol abuse, diabetes mellitus, skin and kidney diseases.^3,4^ The prevalence for non-prosthetic joints is 2 cases/100,000 patient-years.^4^ In a study based on a medical record database in the United Kingdom, the incidence rate of septic arthritis was 0.24 and 0.09 cases per 1000 person-years in individuals with gout and controls with no diagnosis of inflammatory arthritis, respectively.^5^ In multivariate analysis, patients with gout were 2.6 times more likely to be diagnosed with septic arthritis associated with antibiotic use or hospitalisation, compared to controls^5^. In adults, the knee joint is most commonly affected,^2,4^ followed by hip, ankle, elbow, wrist and shoulder.^4^ Treatment with glucocorticoids and a history of rheumatoid arthritis have been associated with polyarticular involvement.^4^

If septic arthritis is suspected, empiric treatment with antibiotics should be immediately initiated.^2,4^ The absence of specific symptoms and blood tests renders arthrocentesis the single most helpful diagnostic tool. A synovial fluid white blood cell (WBC) count > 50,000/cc may indicate septic arthritis,^2,4^ but even lower titres cannot safely rule out the diagnosis.^4^ Furthermore, the existence of MSU crystals in the synovial fluid cannot definitely exclude septic arthritis,^6^ as the septic process may cause crystals to release from synovial membrane or cartilage (“*innocent bystanders*”).^7^ Ultimately, patients with gout should undergo fluid aspiration for Gram’s stain and culture, to rule out possible concomitant septic arthritis.^4^ The sensitivity of Gram’s stain ranges from 29% to 50%, thus a high clinical suspicion and follow-up is very important.^1^

Major causes of non-gonococcal septic arthritis are *Staphylococcus aureus* (approximately 60% of cases) and *Streptococcus* species (∼20%),3,4,9,10 with group B Streptococci accounting for approximately 20% of cases caused by *Streptococcus.*^3^ The incidence of streptococcal septic arthritis has been shown to increase in recent reports.^10^ *Streptococcus agalactiae* is a common microorganism of the *Streptococcus* family (group B), as it can be found in the gastrointestinal and female genitourinary tract and, in addition to arthritis, can cause various infections, such us pneumonia, bacteraemia, endocarditis and urogenital infections.^8^ Gastrointestinal tract ulcers have been considered as a possible portal of entry; interestingly, patient’s #1 hospitalisation was complicated by gastrointestinal ulcer bleeding and these ulcers could potentially preexist.^9^ Polyarticular and oligoarticular septic arthritis by *Streptococcus agalactiae* has also been described in previous literature.^3,4,9,10^ The most common joints involved are the knee, ankle, shoulder and metacarpophalangeal joints. Bacteraemia may be found in 66% of patients with group B streptococcal septic arthritis.^4,10^

Gout is the most common form of inflammatory arthritis,^11,13^ with a prevalence ranging between 0.9%–2.5% of the general population.^12^ It is caused by deposition of MSU crystals in joints and surrounding tissues.^11,13,14^ Treatment of hyperuricemia and gout is important, because untreated disease may lead to tophus formation,^11^ the latter causing bone erosions and joint damage leading to disability.^11^ Apart from chronic arthropathy, chronic hyperuricemia can also cause renal and cardiovascular complications.^14^ According to the EULAR recommendations for management of gout, IL-1 blockers should be considered for treating flares of gout in patients with contraindication, intolerance or non-response to the standard of care medication.^11,12^ Canakinumab, a monoclonal antibody against IL-1β, is approved in Europe for such difficult-to-treat patients.^12^ In patients with refractory gout flares, it has been associated with major improvement in health-related quality of life, especially in physical function.^11^ Considering its high cost, anakinra, an antagonist of IL-1 receptor, may also prove useful as an alternative choice for treatment of gout in difficult-to-treat patients.^12,13^ Janssen et al. showed efficacy of anakinra in treating gout with daily administration for seven days, with no severe safety outcomes.^13^ Patient #1 in our series had a remarkable clinical response initially to anakinra and subsequently to canakinumab.

Reports of concomitant gout and septic arthritis in the literature are scarce and have mainly been reported in case series from Taiwan^7,14^ and Spain.^15^ This coexistence must be considered in patients with coexisting positive cultures and MSU crystals in the synovial fluid from the same joint.^7,14^ Differentiation between septic arthritis and other forms of arthritis, such as crystal-induced or rheumatoid arthritis can be particularly challenging.^1,7,14^ The clinical presentation of gout and septic arthritis may be similar, with marked inflammation, fever and swelling, redness, pain and reduction of range of motion of affected joints.^7,14^ The knee is the most commonly affected joint^7,15^ followed by the ankle and shoulder,^7^ often in an oligoarticular distribution.^14^ In patients with risk factors for septic arthritis, a confident diagnosis of gout should be made only after unequivocal exclusion of infection.^14^ In conclusion, we present two patients with concomitant septic arthritis and gout, highlighting the vicious cycle which is occasionally initiated between these two conditions. A favourable clinical outcome requires prompt diagnosis and effective management of both conditions, often with a combination of antibiotic therapy, surgical drainage and anti-inflammatory medications.
